# Synthesis of Antibacterial Hybrid Hydroxyapatite/Collagen/Polysaccharide Bioactive Membranes and Their Effect on Osteoblast Culture

**DOI:** 10.3390/ijms23137277

**Published:** 2022-06-30

**Authors:** Lucas Fabrício Bahia Nogueira, Marcos Antônio Eufrásio Cruz, Guilherme José Aguilar, Delia Rita Tapia-Blácido, Márcia Eliana da Silva Ferreira, Bianca Chieregato Maniglia, Massimo Bottini, Pietro Ciancaglini, Ana Paula Ramos

**Affiliations:** 1 Departamento de Química, Faculdade de Filosofia, Ciências e Letras de Ribeirão Preto-Universidade de São Paulo, Ribeirão Preto 14040-901, Brazil; lucas.fabricio.nogueira@usp.br (L.F.B.N.); marcos.antonio.cruz@usp.br (M.A.E.C.); guilherme.aguilar@usp.br (G.J.A.); delia@ffclrp.usp.br (D.R.T.-B.); biancamaniglia@usp.br (B.C.M.); pietro@ffclrp.usp.br (P.C.); 2 Department of Experimental Medicine, University of Rome Tor Vergata, 00133 Rome, Italy; massimo.bottini@uniroma2.it; 3 Departamento de Ciências Farmacêuticas, Faculdade de Ciências Farmacêuticas de Ribeirão Preto-Universidade de São Paulo, Ribeirão Preto 14040-900, Brazil; mesfe@fcfrp.usp.br

**Keywords:** collagen, chitosan, hydroxyapatite, hybrid membranes, biomineralization

## Abstract

Inspired by the composition and confined environment provided by collagen fibrils during bone formation, this study aimed to compare two different strategies to synthesize bioactive hybrid membranes and to assess the role the organic matrix plays as physical confinement during mineral phase deposition. The hybrid membranes were prepared by (1) incorporating calcium phosphate in a biopolymeric membrane for in situ hydroxyapatite (HAp) precipitation in the interstices of the biopolymeric membrane as a confined environment (Methodology 1) or (2) adding synthetic HAp nanoparticles (SHAp) to the freshly prepared biopolymeric membrane (Methodology 2). The biopolymeric membranes were based on hydrolyzed collagen (HC) and chitosan (Cht) or κ-carrageenan (κ-carr). The hybrid membranes presented homogeneous and continuous dispersion of the mineral particles embedded in the biopolymeric membrane interstices and enhanced mechanical properties. The importance of the confined spaces in biomineralization was confirmed by controlled biomimetic HAp precipitation via Methodology 1. HAp precipitation after immersion in simulated body fluid attested that the hybrid membranes were bioactive. Hybrid membranes containing Cht were not toxic to the osteoblasts. Hybrid membranes added with silver nanoparticles (AgNPs) displayed antibacterial action against different clinically important pathogenic microorganisms. Overall, these results open simple and promising pathways to develop a new generation of bioactive hybrid membranes with controllable degradation rates and antimicrobial properties.

## 1. Introduction

Lesions caused by both trauma and pathologies may limit the spontaneous self-repair ability of the bone tissue. Critical-size defects may disfavor cell attachment to the extracellular matrix (ECM), affecting mineralization and neo-bone formation [1,2,3,4]. Tissue engineering can overcome this problem through the development of implantable materials that can induce cell differentiation and guide mineralized tissue formation [1,5,6]. Ideally, these processes should result in complete apposition between the implant and bone, favoring osseointegration [1]. In this sense, developing polymeric membranes for guided tissue regeneration (GTR) can be highlighted [7,8,9,10,11].

Polymeric membranes for GTR are widely applied in maxillofacial surgeries, specifically to fill bone defects caused by tumor excision or tooth extraction and to replace spongy bone deficiency for metallic implant installation [7,10,11]. These membranes should be implanted at the bone/defect interface, where they act as a barrier that prevents the growth of other connective tissues and undesirable cell infiltration [7,10,11,12,13]. Moreover, bifunctional polymeric membranes have been developed to promote GTR simultaneously at the bone defect interface, thus favoring new tissue formation through osteoblast adhesion, proliferation, and growth. Polymeric membranes for GTR can also promote controlled delivery of osteogenic compounds at the defect site [7,10,11,13,14].

Producing polymeric membranes that can conduct the cascade of events involved in tissue regeneration requires that the native tissue composition and structure be reproduced, to create an artificial ECM that mimics the cell’s natural microenvironment [5,7,11,12]. In addition to the biochemical stimulus, the polymeric membranes must offer mechanical support and isolate and preserve the defect site by stimulating tissue repair at a controlled biodegradation rate without the need for additional removal surgery at the end of the treatment [1,3,5,10]. In this perspective, bone mineral contributes to the tissue’s mechanical properties and supports the tissue remodeling process and biochemical function as an ionic reservoir [15,16]. This is ascribed to the low degree of crystallinity of the bone minerals, which provides an adequate dissolution/resorption rate.

To date, several strategies have been proposed to produce hybrid membranes for GTR by mixing a previously synthesized mineral phase with a polymeric membrane [7,11,17,18,19,20]. However, producing a biomimetic mineral phase in the hybrid material remains challenging. HAp particles resembling the bone ECM environment cannot be precipitated in the absence of an organic matrix. For this reason, it is crucial to develop innovative strategies to produce hybrid membranes based on guided precipitation of the mineral phase through confined spaces created by the organic matrix [10,20,21].

Compared to synthetic polymers, biopolymers have lower toxicity and higher biodegradability. Furthermore, biopolymers can mimic the ECM constituents and structure, so that they can easily be recognized and enzymatically metabolized by the human body [5,19,22,23]. Using type-I collagen to produce biocompatible, biodegradable, and bioresorbable membranes for GTR is a good example of applying a protein present in the bone organic matrix [7,10,24]. Alternatively, native collagen can be replaced with hydrolyzed collagen (HC). This is advantageous because amino acids are highly bioavailable in HC, which supports collagen biosynthesis, to maintain the cartilage and bone matrix structure [25,26,27,28]. Nevertheless, collagen-based materials, especially HC-based materials, have poor mechanical properties and may undergo rapid biodegradation and resorption due to the enzymatic activity of collagenase produced by macrophages and leukocytes [25,26]. To overcome these issues, the bioactivity and mechanical properties of HC-based biomaterials can be modulated by combining them with other biopolymers. Hence, using blends consisting of two or more biocompatible and bioresorbable biopolymers to produce the membranes is an alternative for improving the mechanical properties and biomedical performance of biopolymeric membranes [11,12,29,30]. Polysaccharides such as chitosan (Cht) and κ-carrageenan (κ-carr) are promising candidates to improve the mechanical properties of HC-based biopolymeric membranes. These molecules share chemical and structural similarities with non-collagenous macromolecules including sulfated and non-sulfated glycosaminoglycans present in the bone ECM [29,31]. Additionally, Cht can be enzymatically biodegraded by lysozyme [7,12,32,33]. κ-carr, a naturally sulfated polysaccharide obtained from certain red seaweeds [6,34,35,36], contains negatively charged functional groups that may bind to or retain growth factors considered essential for cell differentiation [6,34,35,36]. For these reasons, these polysaccharides have been successfully used in the design of biopolymeric membranes for GTR [7,8,35,36,37,38,39].

In general, innovative strategies must be based on the combination of supramolecular self-organization of biopolymeric chains originating from an ordered three-dimensional template with nucleation of inorganic compounds at the surface and in the interstices of this network. In this context, HAp precipitation can be highlighted—HAp shares chemical and structural similarities with the mineral component of human bones and teeth, not to mention that HAp is biocompatible and bioactive and does not elicit inflammatory, toxic, or immunogenic responses [7,10,20,40,41,42].

Although HAp incorporation can enhance the mechanical properties and optimize the surface characteristics of biopolymeric membranes, improved biological responses will also depend on the synergism between the organic and inorganic phases achieved by the methodology adopted for hybrid membrane synthesis.[7,19,20,43,44,45]. We have successfully demonstrated that in situ precipitation of CaCO_3_ particles into carrageenan hydrogels yields membranes with outstanding mechanical properties depending on the availability of Ca^2+^ binding to the polysaccharide [37].

Here, we describe the design of hybrid membranes based on biopolymeric membranes consisting of HC and a polysaccharide (Cht or κ-carr). We obtained the hybrid membranes by two different methodologies: 1—HAp incorporation in the biopolymeric membrane by in situ precipitation, and 2—ex situ HAp precipitation and further incorporation in the biopolymeric membrane. Then, we assessed the behavior of the hybrid membranes obtained by these different methodologies to understand the role played by the organic matrix as physical confinement during hybrid biomimetic ECM production. To improve the biological performance of the hybrid membranes further, we incorporated silver nanoparticles (AgNPs) in the biopolymeric membranes, to provide them with antimicrobial activity [46,47,48,49,50,51,52]. Indeed, in osteoblast cultures, Albers et al. [53] observed that cytotoxicity and antimicrobial activity depend on silver particle dose and size, with nanoparticles being more harmful than microparticles. Thus, evaluating and optimizing both the synthesis process and AgNP incorporation in hybrid membranes is crucial for the intended material to promote antimicrobial activity without triggering a cytotoxic effect in the osteoblasts (i.e., the cells underlying the bone ECM synthesis) [50,51,52].

## 2. Experimental Section

### 2.1. Materials

All the aqueous solutions were prepared with ultrapure dust-free water from a Milli-Q^®^ system (resistivity = 18.2 MΩ cm). Silver nitrate (AgNO_3_, Sigma Aldrich-St. Louis, MI, USA, 99%), sodium boron hydride (NaBH_4_, Sigma-Aldrich, 99%), phosphoric acid (H_3_PO_4_, Synth, Diadema, Brazil, 85%), calcium chloride dihydrate (CaCl_2_·2H_2_O, Synth-Diadema, Brazil, 99.5%), hydrolyzed collagen (HC) (Bioflora manipularium, Ribeirão Preto, Brazil), κ-carr (Gelymar-Santiago, Chile), Cht (low molecular weight, Sigma Aldrich-St. Louis, MI, USA glycerol (Mallinckrodt Chemicals-Phillipsburg, NJ, USA, 99.8%), and ammonium carbonate ((NH_4_)_2_CO_3_, Neon-Suzano, Brazil, Analytical Grade) were used without further purification. The following chemicals were used to prepare the simulated body fluid (SBF): calcium chloride dihydrate (CaCl_2_·2H_2_O, Synth- Diadema, Brazil, 99.5%), sodium chloride (NaCl, Synth-Diadema, Brazil, 99.5%), sodium bicarbonate (NaHCO_3_, Vetec-Duque de Caxias, Brazil 99.5%), potassium chloride (KCl, Vetec-Duque de Caxias, Brazil, 99.0%), sodium phosphate dibasic heptahydrate (Na_2_HPO_4_·7H_2_O, Mallinckrodt Chemicals-Phillipsburg, NJ, USA, 99.5%), hydrochloric acid (HCl, Synth-Diadema, Brazil, 36–38.5%), magnesium chloride hexahydrate (MgCl_2_·6H_2_O, Synth-Diadema, Brazil, 99.0%), sodium sulfate (Na_2_SO_4_, Synth- Diadema, Brazil), and tris-hydroxymethylaminemethane (C_4_H_11_NO_3_, Sigma Aldrich-St. Louis, MI, USA, 99.9%). The procedure is described elsewhere [43,54].

### 2.2. Hydrogel Preparation for Synthesis of Biopolymeric Membranes

The biopolymeric hydrogels were prepared by dissolving 2.5 wt.% HC with 2.5 wt.% Cht or κ-carr in 10 mL of 0.0165 mol·L^−1^ H_3_PO_4_ or 5 mmol·L^−1^ KCl aqueous solution under stirring at 65 °C for 2 h, followed by stirring at room temperature for 12 h. Then, 25 wt.% glycerol was added to the mixture and kept under stirring for 1 h [55]. The biopolymeric hydrogels were transferred to a Teflon plate and dried at 65 °C in an oven for 24 h, for the biopolymeric membranes (HC + Cht) and (HC + κ-carr) to form.

### 2.3. Inorganic Phase Incorporation and Preparation of Hybrid Membranes

Two methodologies were used to incorporate the mineral into (HC + Cht) and (HC + κ-carr), to obtain the hybrid membranes: (1) in situ precipitation of hydroxyapatite (HAp) into the interstices of the biopolymeric membrane, to obtain (HC + Cht_HAp) and (HC + κ-carr_HAp), respectively, or (2) addition of previously synthesized hydroxyapatite (SHAp) particles in the biopolymeric membrane, to obtain (HC + Cht_SHAp) and (HC + κ-carr_SHAp), respectively.

**Methodology 1:** The mineral phase was precipitated into the interstices of the (HC + Cht) or (HC + κ-carr) through the addition of 10 mL of an aqueous mixture containing 0.05 mol·L^−1^ CaCl_2_·2H_2_O and 0.0165 mol·L^−1^ H_3_PO_4_. The Ca/P molar ratio used herein was higher than the 1.67 found in HAp. Excess Ca^2+^ favors interaction between the carrageenan chains, thus enhancing the gelation process [37,56,57]. Ca^2+^, H_2_PO_4_^−^, HPO_4_^2^, and PO_4_^3−^ present in the mixture were the source for precipitation of the mineral phase after the biopolymeric membrane was exposed to NH_3(g)_ and CO_2(g)_, arising from (NH_4_)_2_CO_3(s)_ decomposition, for 45 min in a closed box. Increasing pH led to the formation of the precipitate within the (HC + Cht) or (HC + κ-carr) chain network, as previously reported [55].

**Methodology 2:** SHAp particles were prepared by exposing a mixture containing 0.05 mol·L^−1^ CaCl_2_·2H_2_O and 0.0165 mol·L^−1^ H_3_PO_4_ (similar to method 1) to NH_3(g)_ and CO_2(g)_ in a closed box for 45 min. The precipitate was washed with deionized water, filtered through a Millipore^®^ cellulose ester membrane (0.45-μm pore size), and dried overnight at 100 °C in an oven. Subsequently, 0.1 wt.% SHAp was added to the previously prepared (HC + Cht) or (HC + κ-carr) under stirring for 1 h [7].

For both methods, the biopolymeric membranes were transferred to a Teflon plate and dried at 65 °C in an oven, to produce the hybrid membranes HC + Cht_HAp, HC + κ-carr_HAp, HC + Cht_SHAp, and HC + κ-carr_SHAp. These hybrid membranes were immersed in 10 mL of a 0.2 mol·L^−1^ NaOH solution for 60 min and washed several times in ultrapure water, to remove excess H_3_PO_4_, and further dried in a closed box at room temperature. Table 1 presents the codes for the biopolymeric membranes (HC + Cht) and (HC + κ-carr) (controls) and the hybrid membranes (HC + Cht)_HAp, (HC + κ-carr)_HAp, (HC + Cht)_SHAp, and (HC + κ-carr)_SHAp, obtained after the mineral phase was incorporated into the controls by one of the two methodologies used in this study.

### 2.4. Compositional and Morphological Characterization

The structure of the mineral precipitated in the hybrid membranes (HC + Cht)_HAp, (HC + κ-carr)_HAp, (HC + Cht)_SHAp, and (HC + κ-carr)_SHAp was evaluated by X-ray diffraction (XRD) analysis (Bruker-AXS D5005 diffractometer); Cu–Kα radiation and nickel monochromator at tube voltage of 40 kV and tube current of 30 mA were used. The mineral precipitated in (HC + Cht)_HAp and (HC + κ-carr)_HAp, prepared by Methodology 1, was characterized in the presence of the membrane, whereas SHAp was analyzed before being incorporated into (HC + Cht) or (HC + κ-carr). The XRD data were compared to X-ray diffraction patterns provided by the crystallography open database (COD) with the aid of automated search-match software tools.

Crystallite size was calculated from the reflection peak corresponding to the (300) plane, indexed at 2θ = 32.9°, according to the Scherrer equation (Equation (1)) [57]:

(1) $$L=\frac{K\;\cdot \;\lambda }{\beta \;\cdot \;cos\theta }$$

where λ represents the X-ray radiation wavelength (0.154 nm), θ is the diffraction angle, K = 0.89, and β is the diffraction peak full width at half-maximum in radians [57].

The chemical groups present in (HC + Cht), (HC + κ-carr), (HC + Cht)_HAp, (HC + κ-carr)_HAp, (HC + Cht)_SHAp, and (HC + κ-carr)_SHAp were investigated by Raman spectroscopy between 1400 and 200 cm^−1^; the He/Ne laser excitation source (λ = 632.81 nm) and an optical microscope (Olympus) (MicroRaman HORIBA-Jobin Yvon) were used. The morphological characteristics of (HC + Cht), (HC + κ-carr), (HC + Cht)_HAp, (HC + κ-carr)_HAp, (HC + Cht)_SHAp, and (HC + κ-carr)_SHAp were evaluated by scanning electron microscopy (SEM) under a Shimadzu SS-500 microscope. The samples were coated with a thin gold layer by electrodeposition before analysis.

### 2.5. In Vitro Bioactivity Tests

The bioactivity (i.e., the ability to induce HAp precipitation) of (HC + Cht), (HC + κ-carr), (HC + Cht)_HAp, (HC + κ-carr)_HAp, (HC + Cht)_SHAp, and (HC + κ-carr)_SHAp was investigated after immersion in simulated body fluid (SBF) at 37 °C [43,58,59]. The SBF composition and preparation were previously described by Kokubo and Takadama [43].

The in vitro bioactivity tests were carried out by soaking (HC + Cht), (HC + κ-carr), (HC + Cht)_HAp, (HC + κ-carr)_HAp, (HC + Cht)_SHAp, or (HC + κ-carr)_SHAp, 2.5 × 2.5 cm cutout piece, in 10 mL of SBF at 37 °C for 30 min. After being removed from SBF, the hybrid membranes or controls were exhaustively rinsed with ultrapure water and left to dry at room temperature, in a closed box. The washing procedure ensured that non-specifically adsorbed soluble material precipitated at the hybrid membrane or control surface was dissolved. Finally, the formation of the apatite layer was evaluated by changes in crystallinity and surface morphology of (HC + Cht), (HC + κ-carr), (HC + Cht)_HAp, (HC + κ-carr)_HAp, (HC + Cht)_SHAp, and (HC + κ-carr)_SHAp.

### 2.6. Mechanical Properties

The mechanical properties of (HC + Cht), (HC + κ-carr), (HC + Cht)_HAp, (HC + κ-carr)_HAp, (HC + Cht)_SHAp, and (HC + κ-carr)_SHAp were characterized with the aid of a Texturometer (TA Instrument—TA. TX Plus); the software Texture Expert V.1.22 (SMS) was used. Tensile strength and elongation at break were determined according to the Standard Test Method for Tensile Properties of Thin Plastic Sheeting (ASTM D882-12) [60,61]. The analyses were performed in quintuplicate. For this purpose, membranes with 100 mm length and 6 mm width were cut following the rectangular strips pattern recommended by the ASTM [60,61]. The thickness of each specimen was measured with an electronic digital micrometer (Zaas-Precision).

Before being characterized, (HC + Cht), (HC + κ-carr), (HC + Cht)_HAp, (HC + κ-carr)_HAp, (HC + Cht)_SHAp, and (HC + κ-carr)_SHAp were preconditioned in a desiccator containing a saturated sodium bromide solution (58% RH) for at least 48 h. Then, the hybrid membranes and controls were subjected to tensile tests at a constant rate of 1 mm·s^−1^ starting from an initial separation of 80 mm, until the strip was ruptured. The stress and elongation at rupture were obtained directly from the stress versus strain curves. Young’s modulus (YM) was obtained from the slope of the stress versus strain curve.

### 2.7. Swelling and Degradability

Slices (2.5 × 2.5 cm) obtained from (HC + Cht), (HC + κ-carr), (HC + Cht)_HAp, (HC + κ-carr)_HAp, (HC + Cht)_SHAp, and (HC + κ-carr)_SHAp were weighed (W_ini_) (n = 3) and immersed in 10 mL of phosphate-buffered saline (PBS), pH 7.4, at 37 °C for 1 h. The swollen samples were removed from the solution and rinsed with ultrapure water. Excess water at the surface was removed with absorbent paper, and samples were weighed (W_fin_) (n = 3). Subsequently, to evaluate degradability, the samples were kept in an oven for complete drying under a controlled temperature of 65 °C for 24 h. Then, they were weighed again (W_dried_). The percentages of water absorption (W_a_%) (Equation (2)) and weight loss (W_L_%) (Equation (3)) were calculated by using the following equations:

(2) $$\mathrm{Wa}\%=\left(\frac{\mathrm{Wfin}-\mathrm{Wini}}{\mathrm{Wini}}\right)\times 100$$

(3) $$W_{L}\%=\left(\frac{\mathrm{Wini}-\mathrm{Wdried}}{\mathrm{Wini}}\right)\times 100$$

### 2.8. Wettability and Surface Free Energy

The wettability of (HC + Cht), (HC + κ-carr), (HC + Cht)_HAp, (HC + κ-carr)_HAp, (HC + Cht)_SHAp, and (HC + κ-carr)_SHAp was evaluated by measuring the contact angles (θ) of a water drop spread at the membrane surface with a DataPhysics OCA20 goniometer (software SCA-20) before and after the mineral phase was incorporated. Briefly, the software extracted the drop profile by using the elliptical approach to measure the tangent of the angle formed between the surface and the drop. Surface free energy (SFE) was also determined by using the same procedure for θ measurements of three test liquids (water, diiodomethane, and formamide). SFE was calculated by using the Owens–Wendt–Kaeble equation [62] (Equation (4)):

γ_L_ (1 + cos θ) = 2(γ_L_^d^ γ_S_^d^)^1/2^ + 2(γ_L_^p^ γ_S_^p^)^1/2^ (4)

where subscripts S and L represent the solid and liquid surfaces, respectively, and γ^d^ and γ^p^ correspond to the dispersive and polar component of the total SFE (γ_TOTAL_ = γ^d^+ γ^p^), respectively [63].

### 2.9. Incorporation of Silver Nanoparticles (AgNPs) into the Biopolymeric Membranes

AgNPs were added to HC + Cht and HC + κ-carr at a concentration of 5 mg of AgNPs. We used 100 cm^−2^ of biopolymeric membrane, as follows. AgNPs were synthesized by reducing Ag^+^, generated from 1 mmol·L^−1^ AgNO_3_ aqueous solution, with 2 mmol·L^−1^ NaBH_4_ aqueous solution, at a volume ratio of 1 mL:4 mL, respectively [64,65]. AgNP formation was monitored by UV-Vis absorption spectroscopy, particle size, and zeta potential measurements with the dynamic light scattering (DLS) technique on a Zetasizer Nano ZS instrument (Malvern Instruments). Thus, 5 mL of the AgNP aqueous dispersion was mixed with (HC + Cht) or (HC + κ-carr) with the aid of a magnetic stirrer, at 60 °C. The mixture was then stirred at room temperature for 12 h. In the end, yellowish biopolymeric membranes were obtained. This color is characteristic of the presence of AgNPs. Later, these membranes were used to incorporate the mineral phase for obtaining hybrid membranes.

### 2.10. In Vitro Toxicity to Osteoblast Culture

The classic 3-(4,5-dimethylthiazol-2-yl)-2,5-diphenyltetrazolium (MTT) method was used for the cytotoxicity assays; the protocol described by Mosmann [66] and adapted from de Faria et al. [67] was applied. Briefly, murine osteoblastic cells MC3T3-E1 (ATCC™) at first passage were cultured in alpha minimum essential medium (α-MEM, Gibco) supplemented with 10% fetal bovine serum and 1 vol% penicillin/streptomycin. After reaching confluence, the cells were trypsinized to prepare a suspension of 2 × 10^4^ osteoblasts in 1 mL of α-MEM. The osteogenic medium was achieved by adding ascorbic acid and β-glycerophosphate at 0.28 and 10 mmol·L^−1^ per well, respectively. Later, the suspension was added to a 24-well microplate containing 1 × 1 cm samples cut from the membranes, which was enough to cover the bottom of the wells. The membranes were sterilized before the cultures by exposure to UV radiation (λ = 380 nm) for 40 min (20 min each side). Incubation at 37 °C and 5% CO_2_ was accomplished for 24 and 72 h. Subsequently, 1.0 mg·mL^−1^ MTT solution was added to the medium containing the osteoblasts, followed by incubation at 37 °C for 4 h. During this time, a highly colored compound (formazan) was produced upon reduction with NADH, which reflected the cellular dehydrogenase activity. After incubation for 4 h, the formazan crystals were dissolved in 2-propanol and stirred until complete dissolution. Absorbance was read at 560 and 690 nm on a spectrophotometer (SpectraMaxM3) to determine the mitochondrial dehydrogenase concentration. Osteoblast viability was expressed as the average percentage of viable cells from three experiments as compared to the control (cells cultivated on polystyrene discs—100% viability), on each day of culture.

### 2.11. Antimicrobial Activity Assay

The antimicrobial activity of the hybrid membranes against *Escherichia coli* (*E. coli*), *Staphylococcus aureus* (*S. aureus*), and *Pseudomonas aeruginosa* (*P. aeruginosa*) was investigated by the agar diffusion assay. Bacterial inocula were grown on Tryptone Soya Agar (TSA, Oxoid) plates 24 h before the antimicrobial activity test. Culture suspensions were prepared from a dilution in 0.85 wt% saline, with a turbidity equal to that of a McFarland no. 1 standard (Probac do Brasil), which corresponds to approximately 3 × 10^8^ CFU·mL^−1^. Subsequently, 100 μL of the bacterial suspension was spread on the surface of Petri dishes containing 20 mL of TSA medium, with the aid of a Drigalski spatula. HC + Cht)_HAp and (HC + κ-carr)_HAp, prepared in the absence of AgNPs, were adopted as a control for the tests. The samples were cut into 1-cm diameter discs and sterilized by ultraviolet radiation for 5 min. The discs were pressed against the culture medium inoculated with the suspension of *E. coli*, *S. aureus*, or *P. aeruginosa* and incubated at 37 °C for 24 h. Subsequently, the bacterial inhibition zone was monitored by measuring the difference between the diameter of the clear inhibition zone around the film disc, where bacterial growth is inhibited, and the film disc. Larger inhibition zones correspond to greater antimicrobial activity. Technical and biological replicas of the tests were performed.

### 2.12. AgNP Release from the Membranes In Vitro

AgNP release from (HC + Cht) or (HC + κ-carr) was accompanied by UV-Vis absorption spectrometry on a Hewlett Packard 8453 spectrophotometer. (HC + Cht) and (HC + κ-carr) obtained in the presence and absence (control) of AgNPs were cut into 1-cm^2^ squares and immersed in a quartz cuvette (1-cm optical path) containing 1.5 mL of PBS and kept at 37 °C for 24 h. Changes in the absorbance at the plasmonic resonance band of AgNP (390 nm) were followed at 1, 3, 5, 18, 20, and 24 h. To determine the concentration of AgNPs released by (HC + Cht) or (HC + κ-carr), an external calibration curve was prepared by using the AgNP stock solution.

### 2.13. Statistical Analyses

The data were statistically analyzed by one-way analysis of variance (ANOVA), assuming a confidence level of 95% (*p* < 0.05) for statistical significance. All the data were expressed as means ± standard deviation (SD). In the case of the osteoblast viability assay, statistical comparisons were accomplished by two-way ANOVA followed by Bonferroni’s test for all the data sets.

## 3. Results and Discussion

### 3.1. Composition and Morphology of the Hybrid Membranes

**Figure 1:**
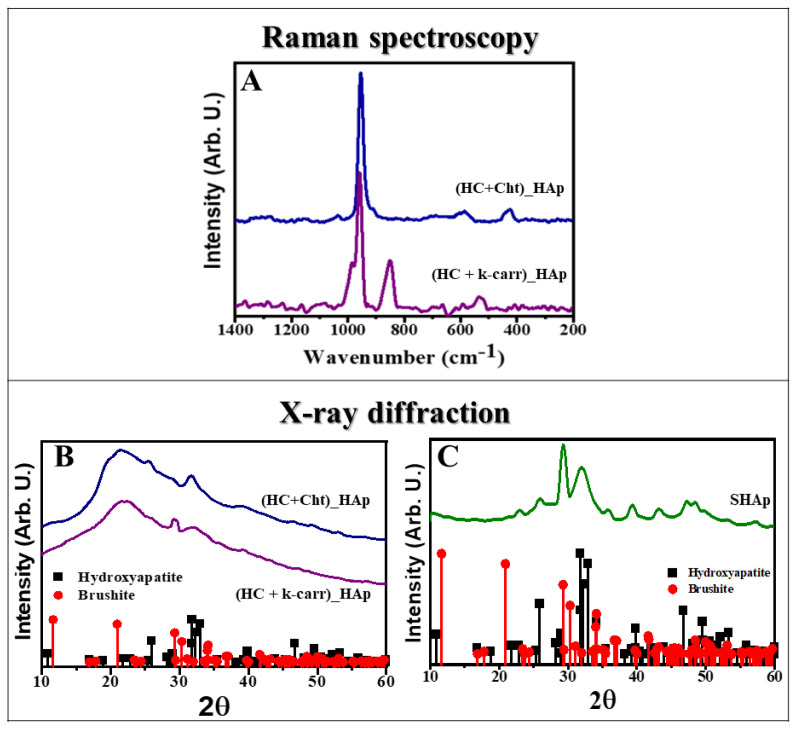
**Structural characterization of the hybrid membranes.** (**A**) Raman spectra of the biopolymeric membranes composed of hydrolyzed collagen (HC) and chitosan (Cht)—blue line or kappa—carrageenan (κ—carr)—purple line after precipitation of the mineral phase by Methodology 1 (HAp). (**B**) X—ray diffraction pattern of the mineral phase precipitated in the presence and (**C**) in the absence of the biopolymeric matrix (SHAp). The mineral phase was identified by the diffraction patterns (■) 9011092—hexagonal hydroxyapatite and (●) 1533075—monoclinic brushite from the Crystallography Open Database (COD).

**Figure 2:**
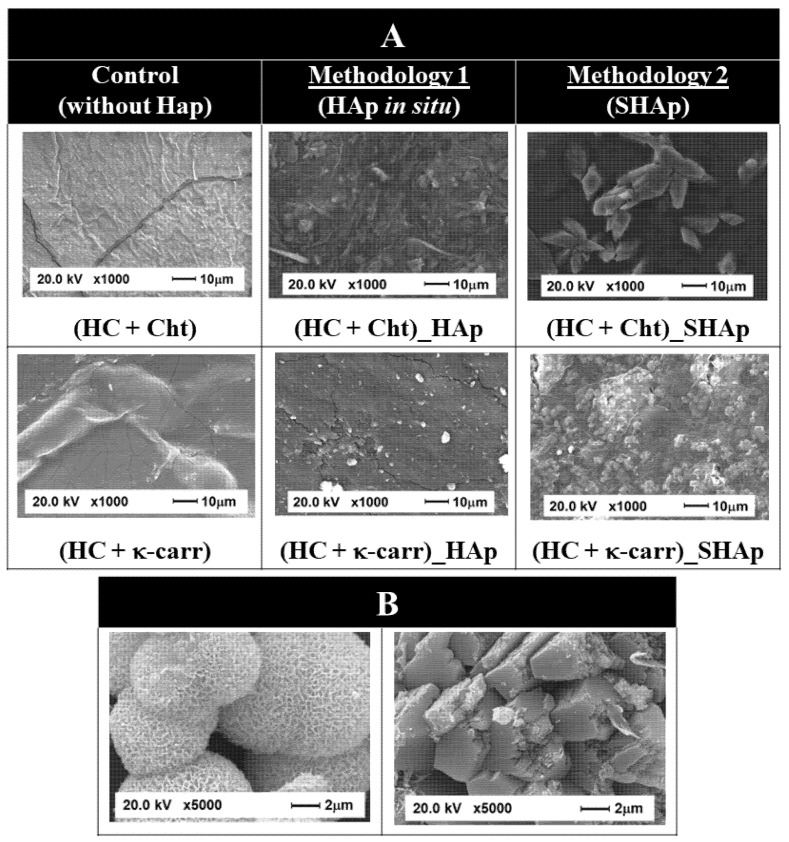
**Morphological characterization of the membranes.** (**A**) SEM images of biopolymeric membranes before (control) and after incorporation of the mineral by Methodology 1 or 2. (**B**) SEM images of SHAp showing different morphologies that can be assigned to the formed HAp (needle-like) and brushite (plate-like).

We characterized (HC + Cht)_HAp and (HC + Cht)_SHAp by Raman spectroscopy, XRD, and SEM (Figure 1 and Figure 2). HAp formation in both (HC + Cht) and (HC + κ-carr) was attested by the presence of an intense band at ~960 cm^−1^ in the Raman spectra (Figure 1A, purple and blue lines), assigned to symmetric stretching of the phosphate group [68]. Less intense bands at ~600 and ~450 cm^−1^ were also observed. The literature relates these low- or medium-intensity bands to other vibrational modes of the HAp structure [69]. The presence of a relatively intense band at 849 cm^−1^ was also observed in the Raman spectrum of (HC + κ-carr)_HAp (Figure 1A, purple line). High intense bands outside the range from 900 to 1000 cm^−1^ can be attributed to vibrational modes from other phosphate minerals simultaneously precipitated with HAp or functional groups present in the structure of the polysaccharides [70,71]. For instance, the Raman spectrum of brushite exhibit an intense broad band centered at 850 cm^−1^, which might support the existence of this mineral phase in the samples precipitated in the presence of (HC + κ-carr) [72].

XRD analysis (Figure 1B) revealed that the mineral phase grown in the interstices of (HC + Cht) and (HC + κ-carr) consisted of HAp, as attested by the intense peaks at 2θ = 26°, 31.8°, and 32.9°, related to the HAp hexagonal phase planes (002), (211), and (300), respectively (COD 9001233), corroborating the data obtained by Raman spectroscopy [57,72]. There was also a broad band at 2θ~20°, related to the amorphous structure of (HC + Cht) and (HC + κ-carr) in the diffraction patterns of both (HC + Cht)_HAp and (HC + κ-carr)_HAp.

The presence of a peak at 2θ = 29 ° observed in the diffractogram of (HC + κ-carr)_HAp (Figure 1B, purple line) supports the formation of brushite into this membrane [71], corroborating the Raman spectrum. This finding makes clear the influence of the membrane composition on the resultant mineral phase, which is important regarding the mineral precipitation induced by the membranes after implantation.

We characterized the crystalline structure of the SHAp particles before adding them to (HC + Cht) or (HC + κ-carr) (Figure 1C). The intense peaks at 2θ = 26° and 31.8° and the less intense peaks at 28.7°, 39.9°, 46.7°, 49.9°, and 53.4° attested the correspondence with the HAp diffraction pattern (COD 9001233). The peak at 2θ = 29 ° revealed simultaneous precipitation of brushite (COD 1533075).

We evaluated whether the morphology of (HC + Cht) and (HC + κ-carr) changed after in situ HAp formation, as well as the morphology of the mineral phase SHAp, precipitated in the absence of the biopolymeric membrane, by SEM. The images in Figure 2 revealed that (HC + Cht) and (HC + κ-carr) presented rough surfaces. Precipitation of the mineral phase modified the surface morphology: a particulate phase that was homogeneously dispersed and embedded in the interstices of the membranes emerged. (HC + Cht) and (HC + κ-carr) provided a 2D template for HAp growth. In these templates, functional groups, such as −NH_2_, can electrostatically interact with phosphate ions, while −OSO_3_^−^ can bind to Ca^2+^, which is essential for HAp nucleation and growth [57]. Phosphate and Ca^2+^ diffuse more slowly through the membrane, increasing the local supersaturation and promoting nucleation in the interstices of the membrane, which act as a 3D-confined medium for mineral growth. Particle growth is controlled by the diffusion of the ions from inside the membrane to its surface [57]. The average size of HAp crystallites precipitated in the presence of Cht or κ-carr was 12 and 13 nm, respectively, as calculated by the Scherrer equation. These values were in the same size range reported for human bone apatite [41,57,73].

Regarding the morphology of the SHAp particles, the SEM images in Figure 2 revealed the formation of particles with two different morphologies—needle-like and plate-like—corroborating the formation of HAp and brushite, respectively, as evidenced by XRD analysis. The needle-like morphology incorporated in spherical structures is common for HAp, while the plate-like morphology is characteristic of brushite [73,74,75,76,77].

**Figure 3:**
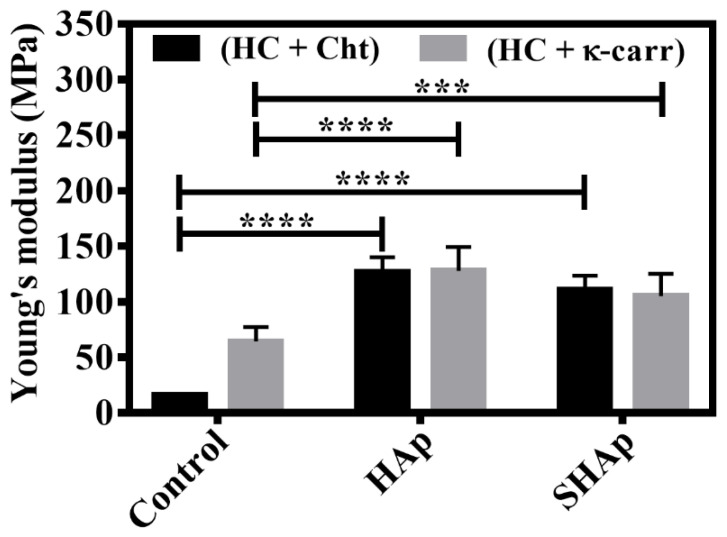
**Mechanical properties of the biomembranes.** Comparison of the Young’s modulus obtained for the membranes based on hydrolyzed collagen (HC) and chitosan (Cht), referred to as control, ((HC + Cht), (HC + Cht)_HAp, and (HC + Cht)_SHAp) and on hydrolyzed collagen (HC) and κ-carrageenan (κ-carr) ((HC + κ-carr), (HC + κ-carr)_HAp, and (HC + κ-carr)_SHAp). Results represent the mean + SD for quintuplicate determination for one experiment. Multiple statistical comparisons were performed by two-way ANOVA in relation to the control, **** *p* < 0.0001 and *** *p* = 0.0008.

Changes in surface morphology assigned to the presence of nanoparticles were clear in the SEM images of (HC + Cht)_SHAp and (HC + κ-carr)_SHAp. In these hybrid membranes, there was heterogeneous dispersion of a particulate phase embedded in the membranes, which contrasted with the homogeneous dispersion of the HAp particles obtained after in situ precipitation. These data support the higher YM found for (HC + Cht)_HAp and (HC + κ-carr)_HAp (Figure 3).

### 3.2. Mechanical Properties

Analyzing the mechanical behavior of biopolymeric membranes, especially determining whether they are resistant in a biological medium, is key for biomedical application. Here, we determined the mechanical properties of the hybrid membranes without ((HC + Cht) and (HC + κ-carr), control) and with the mineral phase incorporated by using Methodology 1 ((HC + Cht_HAp) and (HC + κ-carr_HAp), in situ) or 2 ((HC + Cht_SHAp) and (HC + κ-carr_SHAp), ex situ). Figure 3 shows that (HC + κ-carr) was more rigid (YM: 70 MPa) than (HC + Cht) (YM: 18 MPa). The higher YM of (HC + κ-carr) can be assigned to the electrostatic interaction between the Ca^2+^ and sulfate groups present in the polysaccharide structure, which promoted self-organization of the chains and formed a 3D network [56,57]. The YM we found for (HC + Cht) was close to the values reported [8] for membranes consisting exclusively of Cht. 

Figure 3 also shows that mineral phase incorporation increased the stiffness of the hybrid membranes resulting from (HC + Cht) and (HC + κ-carr). The mechanical properties of 3D-polymeric matrixes are usually optimized by the addition of uniformly dispersed nanoparticles throughout the matrix—the inorganic and organic components establish physical and chemical interactions [21]. Lee et al. [8] observed that silica addition to Cht membranes increased the YM from 15 to 61 MPa. Jegal et al. [21] also described increased YM for poly(lactide-co-caprolactone) and gelatin blends after the addition of apatite nanoparticles. Teng et al. [78] observed that the addition of 20 wt.% HAp to membranes containing 1 wt.% Cht increased the YM from 7 to 1303 MPa. These authors also reported that larger amounts of HAp, greater than 20 wt.%, in the membrane reduced the YM due to disruption of the intermolecular interactions between the Cht polymeric chains [78].

Although (HC + Cht) had lower YM than (HC + κ-carr), (HC + Cht_HAp) and (HC + Cht_SHAp) had improved stiffness, with YM resembling the YM obtained for (HC + κ-carr). In other words, (HC + Cht_HAp) and (HC + Cht _SHAp) seemed to respond in the same way as (HC + κ-carr), as judged by the fact that the YM values were not statistically different. However, the presence of different polysaccharides (Cht or κ-carr) in the hybrid membrane composition caused distinct increases in the YM value in relation to the corresponding control. Therefore, even though (HC + Cht)_HAp, (HC + κ-carr)_HAp, (HC + Cht)_SHAp, and (HC + κ-carr)_SHAp, presented the same final behavior in terms of YM, (HC + Cht) and (HC + κ-carr) were organized differently. HAp incorporation in (HC + Cht) increased YM by 550% and 475% in (HC + Cht)_HAp and (HC + Cht)_SHAp, respectively, whereas HAp incorporation in (HC + κ-carr) increased YM by 85.7% and 57.1% in (HC + κ-carr)_HAp and (HC + κ-carr)_SHAp, respectively. (HC + Cht)_HAp and (HC + κ-carr)_HAp presented the highest YM, 130.1 ± 15.9 and 133 ± 24 MPa, respectively.

(HC + Cht)_HAp and (HC + Cht)_SHAp, containing Cht, showed the most significant increases in YM, evidencing more significant incorporation of the mineral phase in (HC + Cht), mainly in (HC + Cht)_HAp, obtained by Methodology 1. We prepared (HC + Cht)_HAp at pH close to 2.7, in which both the Cht chains and HC residues were positively charged, which hindered attractive electrostatic interactions. However, when we increased the pH for mineral precipitation, the HC residues became negatively charged due to deprotonation of the carboxylic groups, which then electrostatically interacted with the positively charged Cht amino groups. Thus, the formation of this polyelectrolytic complex resulted in an organized biopolymeric chain with significant synergism between the organic and inorganic phases, increasing the resistance. This sequence of events justified the fact that the YM of (HC + Cht)_HAp increased by around 130% in relation to (HC + Cht), while for (HC + κ-carr)_HAp, the increase was close to 115% in relation to (HC + κ-carr). In the latter case, interactions between the calcium ions and the sulfate groups present in the κ-carr polymeric chains affected the mineral phase formation in the interstices of (HC + κ-carr), resulting in lower synergism between the organic and inorganic phases and a thus smaller increase in YM compared to (HC + Cht)_HAp.

Here, incorporation of the mineral phase in (HC + Cht) and (HC + κ-carr) by using Methodology 1 or 2 increased YM to values within the range reported for cancellous bone (50–500 MPa) [79]. Comparing the YM results (Figure 3), (HC + Cht)_SHAp and (HC + κ-carr)_SHAp had a smaller increase in YM than (HC + Cht)_HAp and (HC + κ-carr)_HAp. This distinction might be related to the more heterogeneous distribution of the mineral phase through the membrane and to the excessive amount of HAp in (HC + Cht)_SHAp and (HC + κ-carr)_SHAp. Indeed, Teng et al. [78] reported that interruption of intermolecular interactions due to excess mineral phase leads to lower YM.

Other mechanical parameters such as tensile strength and elongation at break are presented in the supplementary material (Figure S1). In general, the type of polysaccharide (Cht or κ-carr), presence of HAp, and methodology applied for mineral phase incorporation in (HC + Cht) and (HC + κ-carr) strongly influenced the mechanical properties of the membranes. For all the membranes, HAp incorporation increased the tensile strength and decreased the elongation at the break regardless of the employed HAp incorporation methodology. Besides the reinforcing effect, maintaining a high degree of flexibility should favor the use of these hybrid membranes in bone tissue regeneration. Although YM was in the range observed for spongy bone, (HC + Cht)_HAp, (HC + κ-carr)_HAp, (HC + Cht)_SHAp, and (HC + κ-carr)_SHAp had low elongation rate (~30%), corroborating that the most rigid material is also the least stretchable.

### 3.3. Physical-Chemical Properties: Surface Wettability, Surface Free Energy, and Water Absorption Ability

Wettability and surface free energy may dictate how proteins and cells will interact with the surface of an implanted material, further controlling osseointegration [80]. We evaluated the hydrophilicity of (HC + Cht), (HC + κ-carr), (HC + Cht)_HAp, (HC + κ-carr)_HAp, (HC + Cht)_SHAp, and (HC + κ-carr)_SHAp by measuring the contact angle (θ) formed between the surfaces and water droplets (Table 2). We also measured θ by using liquids with different polarities and used the values to calculate the free surface energy (γ_S_) and their respective polar (γ_S_^P^) and dispersive (γ_S_^D^) components, which are shown in Table 2.

(HC + Cht) and (HC + κ-carr) showed θ values of 72.38 ± 8.16 and 58.99 ± 7.66, respectively, in agreement with literature data [18,81]. The high value observed for (HC + Cht) reflected the presence of hydrophobic groups, such as acetylenes, in the chemical structure of the repetitive unit of this polysaccharide.

These values also reflected the higher water absorption ability (%W_a_) of (HC + κ-carr) in PBS. In general, the %W_a_ of a material depends on the presence of hydrophilic chemical groups in the polysaccharide structure, among other properties. κ-Carr is a strong polyelectrolyte and highly soluble polysaccharide in the whole pH range due to the negatively charged sulfate groups [82]. In turn, Cht is poorly soluble in neutral and alkaline solutions due to NH group deprotonation [32]. As shown in Table 2, while (HC + κ-carr) had W_a_= 611%, (HC + Cht) presented W_a_= 143%, a result of its lower hydrophilicity [7,83]. Incorporation of the mineral phase reduced θ for both (HC + Cht) and (HC + κ-carr). Mineral dispersion through the organic matrix added new polar functional groups, such as the HAp hydroxyl, to the membrane, increasing its hydrophilicity [13]. This is important for membranes intended for bone regeneration given that the body fluid is predominantly polar, so hydrophilic surfaces should favor interactions with a physiological medium [43,45,54,84,85].

Likewise, incorporation of the mineral phase increased %W_a_ for (HC + Cht)_HAp, (HC + κ-carr)_HAp, (HC + Cht)_SHAp, and (HC + κ-carr)_SHAp (Table 2). Regarding the methodology used to produce the hybrid membranes, (HC + Cht_HAp) and (HC + κ-carr_HAp), obtained by in situ HAp precipitation, exhibited the greatest reduction in θ and increase in %W_a_ in relation to (HC + Cht) and (HC + κ-carr). Therefore, controlled precipitation guided by the organized structure of (HC + Cht) and (HC + κ-carr) favored the formation of hybrid membranes with mineral particles homogeneously distributed in the organic matrix (Figure 2). According to Caridade et al. [18], enhanced superficial interaction between the organic and inorganic phases may facilitate water penetration through the membrane. In addition, precipitation of the mineral phase in the interstices of (HC + Cht) and (HC + κ-carr) might result in a different HAp concentration in the hybrid membrane as compared to the HAp concentration in the hybrid membranes obtained by Methodology 2 (ex situ HAp precipitation).

However, there were no significant differences in the %W_a_ of (HC + κ-carr) and (HC + κ-carr)_HAp. The smaller influence of mineral addition on %W_a_ could be assigned to the fact that there were fewer Ca^2+^ to precipitate as a mineral because these ions were bound to the κ-carr helical structure, affecting the formation and dispersion of the mineral in the biopolymeric matrix [17,36].

The parameter γ_S_ is also related to the chemical groups present on the membrane surface. In this perspective, this parameter, as well as θ and %W_a_ can predict how the polar and non-polar groups of proteins and cell membranes will interact with different surfaces [86]. Table 2 shows that the polysaccharide type did not result in a significant difference in γ_S_, in agreement with the literature [37,87]. However, the presence of different chemical groups in the polysaccharide structure influenced γ_S_^P^. Supporting their higher hydrophilicity, (HC + κ-carr)_HAp and (HC + κ-carr)_SHAp showed greater contribution from this component to the total γ_S_ as compared to (HC + Cht)_HAp and (HC + Cht)_SHAp.

As for the different methodologies used in this study, as already observed during the analysis of the parameters θ and %W_a_, incorporation of the mineral phase significantly increased γ_S_ as compared to the controls. In general, (HC + Cht)_HAp, (HC + κ-carr)_HAp, (HC + Cht)_SHAp, and (HC + κ-carr)_SHAp had increased γ_S_ and γ because, in contrast to (HC + Cht) and (HC + κ-carr), there was a greater contribution from γ_S_^P^ to γ_S_. For (HC + Cht) _HAp and (HC + Cht)_SHAp, γ_S_ and γ increased by around 165% and 85% in relation to (HC + Cht), respectively, whereas for (HC + κ-carr)_HAp and (HC + κ-carr)_SHAp, γ_S_ and γ increased by 182% and 79% in relation to (HC + κ-carr), respectively. These values were related to variations in the chemical composition of the hybrid membrane surface, which depended on the polysaccharide type and methodology used to incorporate the mineral phase into (HC + Cht) and (HC + κ-carr).

Comparing the methodologies adopted herein, (HC + Cht)_HAp showed more significantly increased surface parameters, which might be related to the dispersion of the mineral phase through the membrane. The same behavior was not observed for (HC + κ-carr)_HAp and (HC + κ-carr)_SHAp because, once again, the low availability of Ca^2+^ affected both the formation and dispersion of the mineral phase in the membrane, indicating that the surface chemical composition and organization of the hybrid membranes were similar.

In general, surface changes that lead to a balance between the non-polar and polar components of γ_S_ are desirable in materials for biomedical applications. The polar interactions with the host tissue favor contact with the body fluid, while the non-polar interactions favor the adhesion of proteins that are important for the first osseointegration stages [5,80]. To support the findings concerning the improved mechanical and surface properties of the membranes after the mineral phase was added, we also characterized the membranes with respect to their composition and morphology.

### 3.4. In Vitro Bioactivity Evaluation of the Hybrid Membranes 

**Figure 4:**
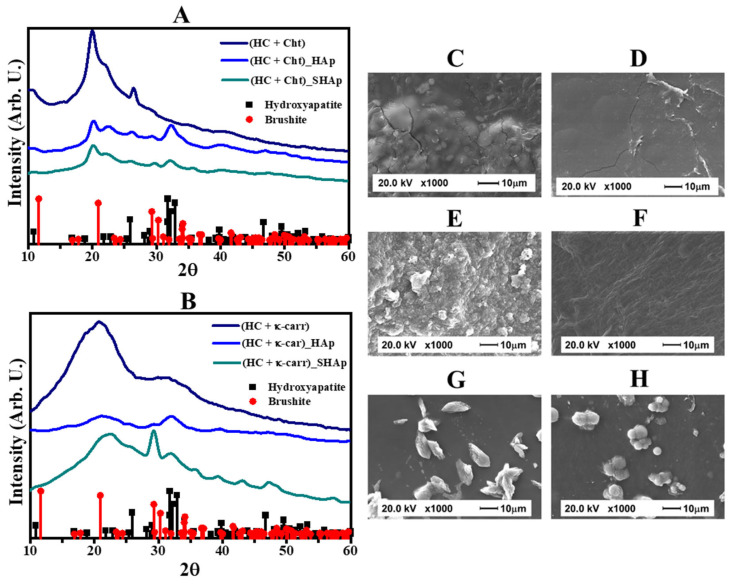
**Characterization of the membranes after the bioactivity test carried out by immersion in SBF.** X-ray diffraction patterns of the HC-based membranes mixed with (**A**) chitosan (Cht), control—dark blue line, (HC + Cht)_Hap—purple line, (HC + Cht)_SHAp—green line, and (**B**) κ-carrageenan (κ-carr), control—dark blue line, (HC + κ-carr)_Hap—purple line, (HC + κ-carr)_SHAp—green line. The mineral phases were identified by the diffraction patterns (■) 9011092-hexagonal hydroxyapatite and (●) 1533075-monoclinic brushite from the Crystallography Open Database (COD). SEM images of biopolymeric membranes after exposure to SBF for 30 min (**C**) (HC + Cht) and (**D**) (HC + κ-carr) (controls); (**E**) (HC + Cht)_HAp; (**F**) (HC + κ-carr)_HAp; (**G**) (HC + Cht)_SHAp; and (**H**) (HC + κ-carr)_SHAp.

We evaluated the bioactivity of (HC + Cht), (HC + κ-carr), (HC + Cht)_HAp, (HC + κ-carr)_HAp, (HC + Cht)_SHAp, and (HC + κ-carr)_SHAp in vitro by immersion in SBF. HAp formation after this procedure indicates bioactivity [43]. We monitored changes in membrane composition by XRD. Figure 4A,B brings the diffractograms (HC + Cht) and (HC + κ-carr) after immersion in SBF for 30 min. The presence of a broad peak at 2θ~20°, related to the (HC + Cht) and (HC + κ-carr) amorphous structure, and the absence of defined peaks in the diffraction pattern of (HC + Cht) and (HC + κ-carr) evidenced their low bioactivity (Figure 4A,B—dark blue lines). This finding corroborated the SEM images (Figure 4C,D), which evidenced no mineral formation in (HC + Cht) or (HC + κ-carr) exposed to SBF.

For (HC + Cht)_HAp and (HC + κ-carr)_HAp, the diffractograms obtained after immersion in SBF for 30 min (Figure 4A,B—purple and green lines) exhibited well-defined peaks corresponding to the HAp structure. For (HC + Cht)_SHAp and (HC + κ-carr)_SHAp (Figure 4A,B—green lines), besides the peaks at 2θ = 26° and 31.8°, the diffractograms displayed less intense peaks at 28.7°, 39.9°, 46.7°, 49.9°, and 53.4 ° after immersion in SBF for 30 min. Figure S2 brings the comparison of the diffraction patterns obtained for the membranes before and after immersion into SBF. Although the direct comparison is hindered by the swelling and partial dissolution of the membranes, the diffraction patterns obtained for the hybrid membranes after exposition to SBF depicted in Figure S2, revealed higher intensity and better definition of the peaks related to the formation of HAp (i.e., θ = 31.8°), in special for the samples obtained by Methodology 1 (Figure S2A,B). Moreover, the relative intensity of the peaks related to the formation of brushite compared to HAp was reduced after the immersion of the membrane (HC + Cht)_SHAp into SBF (Figure S2C).

Comparison of the micrographs of (HC + Cht) and (HC + κ-carr) (Figure 4C,D) with the micrographs of (HC + Cht)_HAp, (HC + κ-carr)_HAp, (HC + Cht)_SHAp, and (HC + κ-carr)_SHAp (Figure 4E–H) after immersion in SBF revealed differences in surface morphology, characterized by deposition of a particulate mineral phase only on the surface of the hybrid membranes. By comparing the SEM images of the hybrid membranes before (Figure 2) and after immersion in the SBF (Figure 4), the amount of particles deposited on the surface of (HC + Cht)_HAp, (HC + κ-carr)_HAp, (HC + Cht)_SHAp, and (HC + κ-carr)_SHAp increased as compared to (HC + Cht) and (HC + κ-carr). The hybrid membranes produced herein did not require longer immersion periods in SBF to stimulate HAp formation, which differed from the description in the study by Mota et al. [7], which described immersion in SBF for at least five days to induce apatite formation. Together, the XRD and SEM results obtained here attested the in vitro bioactivity of (HC + Cht)_HAp, (HC + κ-carr)_HAp, (HC + Cht)_SHAp, and (HC + κ-carr)_SHAp, highlighting the need for the presence of the mineral phase in the composition of the membrane to stimulate apatite deposition.

### 3.5. Biological Properties of the Membranes

#### 3.5.1. Osteoblasts Culture

Understanding the interactions of osteoblasts with biomaterials dedicated to bone repair is important for predicting their biological performance in vivo [88]. Therefore, we conducted cell viability assays to evaluate the influence of biopolymeric membranes on osteoblast proliferation.

**Figure 5:**
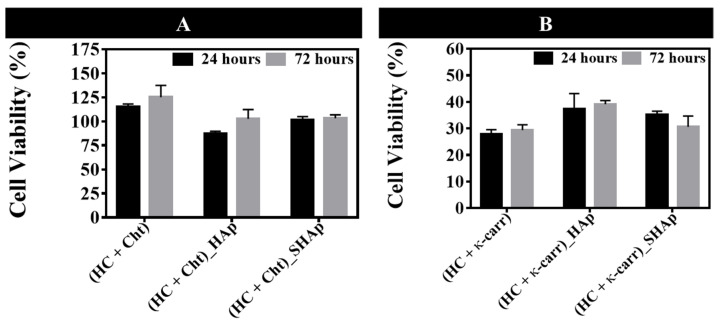
**Effect of the membranes on in vitro osteoblasts culture.** Osteoblast cell viability (as compared to the control: cells cultured on polystyrene discs) in the presence of the membranes based on hydrolyzed collagen (HC) and (**A**) chitosan (Cht) ((HC + Cht), (HC + Cht)_HAp, and (HC + Cht)_SHAp), and hydrolyzed collagen (HC) and (**B**) κ-carrageenan (κ-carr) ((HC + κ-carr), (HC + κ-carr)_HAp, and (HC + κ-carr)_SHAp), containing 5 mg of AgNPs per 100 cm^2^ of membrane.

On the basis of Figure 5A, membranes containing Cht were not toxic to osteoblasts, as indicated by the cell viability values. However, membranes containing κ-carr (Figure 5B) were cytotoxic and reduced cell viability after culture for 72 h. Compared to (HC + κ-carr), the presence of HAp in the membrane did not affect cell proliferation, regardless of the methodology used to prepare the hybrid membrane. This behavior differed from the behavior reported in the literature [7], which describes increased cell proliferation in hybrid membranes as compared to membranes obtained in the absence of the inorganic phase.

Control over the swelling and degradation properties of hydrogels reticulated by intermolecular interactions (such as ionic interactions, hydrophobic interactions, and hydrogen bonds) is associated with the ability of water to penetrate through the matrix, which can disrupt intra- and intermolecular interactions [5,89]. In this study, membranes containing κ-carr were stabilized by ionic interactions, which involved interactions between Ca^2+^ and sulfate groups. κ-Carr is unstable due to its swelling properties and higher degradation, assigned to the uncontrollable exchange of ions with other cations in the surrounding physiological medium. This affects membrane stability even after a mineral phase is added, culminating in lower cell viability.

Considering these aspects, we evaluated in vitro degradation of the membranes by calculating weight loss (%WL) after their immersion in PBS, pH 7.4, at 37 °C for 60 min. The %WL calculated for (HC + Cht) and (HC + κ-carr) was 59% and 64%, respectively, corroborating the data reported by Goonoo et al. [5] and Khoshakhlagh et al. [90]. Here, HC addition may have contributed to higher in vitro degradation of the membranes given the high solubility of this denatured protein in water and polar fluids reported in the literature [28]. Khoshakhlagh et al. [90] observed that the addition of an inorganic phase to a matrix consisting of Cht increased the chemical stability of the hybrid material in PBS as compared to the pure organic matrix. In fact, in our study, we observed this behavior only for (HC + Cht)_HAp, which had WL = 54% and is characterized by homogeneous dispersion of the mineral phase in the interstices of the membrane. However, we did not observe this effect for (HC + κ-carr)_HAp that presented a WL = 65%, similar to the value found for the membrane composed of the pure organic matrix. As already mentioned, ionic interactions between Ca^2+^ and sulfate groups present in each repetitive unit of κ-carr confer low stability to the cross-linked biopolymer.

One of the main functions of biopolymeric membranes applied for GTR is to serve as a matrix to support and to facilitate cell growth and differentiation at the bone defect site, providing support for the structuring of the new tissue [18,35,91]. Thus, in case degradation takes place, it should not interfere with cell growth [35,91]. However, osteoblast proliferation in the presence of the membranes containing κ-carr must have been negatively affected by the low stability and integrity of the biopolymeric matrix in the medium in which the cells were cultured. Therefore, membranes containing Cht in their composition proved to be more advantageous in terms of degradation and, consequently, cell viability, especially (HC + Cht)_HAp.

#### 3.5.2. Antimicrobial Activity of the Membranes in the Presence of Silver Nanoparticles (AgNPs)

We followed AgNP formation by UV-Vis absorption spectroscopy and characterized the resulting nanoparticles by particle size and zeta potential. AgNPs were measured between 3 and 8 nm and had an absorption band at 390 nm and zeta potential of −36 ± 17 mV, which confirmed their colloidal stability (see Supporting Information S1)

Although the AgNP antimicrobial effect has been widely described [50,92], concentration is a very important factor when it comes to the action of these nanoparticles against antibiotic-resistant bacteria. When incorporating AgNPs in materials designed for biomedical applications, one must consider not only the minimum AgNP concentration needed for the antimicrobial activity but also the AgNP safety to mammalian cells. [93] On the basis of the study by Wu et al. [93], who synthesized antibacterial cellulose membranes containing 15 to 45 mg AgNP per 100 cm^2^ and showed that these membranes were not cytotoxic to osteoblasts, here we used 5 mg of AgNPs per 100 cm^2^ of membrane, confirming the absence of cytotoxicity in the concentrations previously reported. 

**Figure 6:**
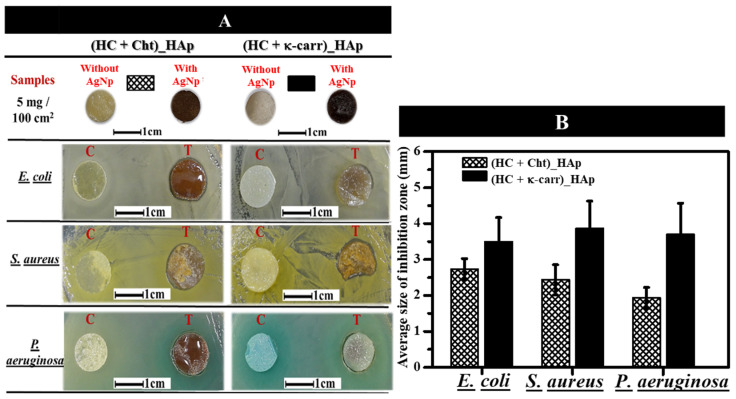
**Antibacterial activity of the membranes.** (**A**) Photography of the hybrid membranes (HC + Cht)_HAp or (HC + k-carr)_HAp in the absence (C) and in the presence (T) of 5 mg of AgNPs per 100 cm^2^. Formation of a halo close to the samples in the presence of *E. coli*, *S. aureus,* and *P. aeruginosa* can be observed after incubation for 24 h. (**B**) Size of the inhibition zone for the hybrid membranes containing AgNPs in the presence of *E. coli*, *S. aureus,* and *P. aeruginosa*.

We carried out the tests by using (HC + Cht)_HAp and (HC + κ-carr)_HAp due to their better dispersion of the mineral phase, smaller θ, higher mechanical resistance, and higher γ_s_ and γ_s_^P^ (factors that favor their application as temporary GTR systems). AgNP incorporation in (HC + Cht) and (HC + κ-carr) (Figure 6) changed the color of the membranes from yellowish to brown. Homogeneous color distribution attested to homogeneous AgNP dispersion through the matrix. We evaluated the antibacterial activity of AgNP-(HC + Cht)_HAp and AgNP-(HC + κ-carr)_HAp (T) and (HC + Cht)_HAp and (HC + κ-carr)_HAp without AgNPs, as a positive control (C), against *E. coli*, *S. aureus*, and *P. aeruginosa* (common bacteria of wound infection) by the agar diffusion assay after incubation at 37 ^o^C for 24 h (Figure 6). AgNP-(HC + Cht)_HAp and AgNP-(HC + κ-carr)_HAp developed an inhibition zone with a diameter ranging from 1.9 to 3.9 mm against the three tested bacteria (Figure 6). As we did not observe an inhibition zone for (HC + Cht)_HAp or (HC + κ-carr)_HAp without AgNPs (C), the antibacterial activity can be assigned only to the presence of AgNPs. The diameter of the inhibition zone resembled the inhibition zone diameter observed by Wu et al. [93] when they used membranes containing about 45 mg of AgNPs per 100 cm^2^. Here, we verified the formation of an inhibition zone by using a lower concentration (i.e., 5 mg of AgNPs per 100 cm^2^ of membrane) than the minimum described by Wu et al. [93] The difference might be related to nanoparticles size. The synthesis of smaller nanoparticles in our study might have favored significant antibacterial action at lower concentrations due to the higher surface area/volume, improving the capacity of these nanoparticles to penetrate the bacterial cell membrane.

Differences in the antibacterial activities of AgNPs against different bacteria are usually assigned to the zeta potential of the particles, which dictates interaction with the cell membrane of Gram-positive and Gram-negative species [51,93]. However, here the surface charge factor was not preponderant because we used the same type of AgNPs exhibiting negative zeta potential for the tests (See Supplementary material, Figure S3). AgNP-(HC + Cht)_HAp gave inhibition zones with a smaller average diameter than AgNP-(HC + κ-carr)_HAp, which is relevant because the larger inhibition zone indicates higher antibacterial activity. The differences may be attributed to changes in the rate of AgNP release by the hybrid membranes. To investigate AgNP release, we immersed the hybrid membranes containing 5 mg of AgNPs per 100 cm^2^ of membrane in PBS at 37 °C and measured changes in the intensity of the UV-Vis absorption band at 390 nm as a function of time. The results obtained after immersion for 24 h (data shown in the supplementary material S2) revealed that AgNP-(HC + Cht)_HAp exhibited a lower rate of AgNP release (1.8%) than AgNP-(HC + κ-carr)_HAp (8.7%). Usually, a slower release rate is advantageous because it may result in an antibacterial effect while preventing cytotoxic effects on human cells. 

Hybrid membranes containing κ-carr exhibited a high degradation rate as described in the previous sections, which supports the augmented AgNP release from AgNP-(HC + κ-carr)_HAp compared to AgNP-(HC + Cht)_HAp. This set of data justifies the large inhibition zones against the bacteria provided by AgNP-(HC + κ-carr)_HAp. With regard to the application of AgNP-(HC + κ-carr)_HAp in bone regeneration, its low stability and the higher rate of κ-carr degradation in fluids with predominantly polar characteristics might result in a cytotoxic effect on osteoblasts due to uncontrolled AgNP release. The set of data indicated that AgNP-(HC + Ch)_HAp is suitable for the development of bone regeneration membranes through osteoblast stimulation and with the possibility of preventing bacterial growth at the tissue/implant interface [51].

## 4. Conclusions

We prepared membranes containing polysaccharides obtained from renewable sources and apatite by two methodologies (for mineral incorporation) and characterized them. In situ precipitation (Methodology 1) was inspired by the natural mineralization of collagen observed in vivo. In turn, the addition of pre-synthesized HAp (Methodology 2) to polymeric blends is a well-known and feasible method to produce organic–inorganic membranes. The presence of minerals improved the mechanical performance of the membranes regardless of the mineral incorporation methodology. The presence of minerals also improved the membrane surface properties related to increased wettability and surface free energy. The XRD, Raman, and SEM analyses revealed that the hybrid membranes produced by Methodology 1 (in situ) resulted in membranes with organized and homogeneous morphology and higher selectivity for exclusive HAp precipitation after exposure to SBF for 30 min. Altogether, Methodology 1 proved to be more advantageous. In fact, slow HAp precipitation guided by the organized structure of the biopolymeric membrane favored greater interaction between the organic and inorganic phases, with the addition and homogeneous dispersion of new polar groups on the surface. Moreover, there were in the physical-chemical and mechanical properties depending on the composition of the biopolymeric membrane used in the synthesis of the hybrid membranes. In fact, different amounts of HAp were formed in the presence of Cht and κ-carr, which may be higher in the presence of Cht compared to κ-carr due to the interactions of the sulfate groups with Ca^2+^ in the latter case.

Membranes containing Cht were not toxic to osteoblasts, whereas membranes containing κ-carr reduced cell viability after culture for 24 and 72 h. This could be related to the higher degradation rate, low stability, and low integrity in the cell culture medium of the membranes containing κ-carr. After AgNPs were incorporated into the membranes, membranes produced by Methodology 1 presented antibacterial action against *E. coli*, *S. aureus*, and *P. aeruginosa*, and this action depended on the AgNP release rate. The set of data suggested that the hybrid membranes composed of HC and Cht and obtained by in situ HAp precipitation can be potentially used as antibacterial membranes, paving the way for their application as bone-repair membranes given that AgNPs do not affect osteoblast viability.

## Figures and Tables

**Table 1:** Codes for the hybrid membranes prepared by using hydrolyzed collagen (HC) and chitosan (Cht) or kappa-carrageenan (κ-carr) and hydroxyapatite (HAp) precipitation in situ (Methodology 1) or ex situ (Methodology 2).

Membrane/Components		HC	Cht	κ-carr	HAp In Situ	SHAp
**Control**	**(HC + Cht)**	✓	✓	0	0	0
	**(HC + κ-carr)**	✓	0	✓	0	0
**Methodology 1**	**(HC + Cht)_HAp**	✓	✓	0	✓	0
	**(HC + κ-carr)_HAp**	✓	0	✓	✓	0
**Methodology 2**	**(HC + Cht)_SHAp**	✓	✓	0	0	✓
	**(HC + κ-carr)_SHAp**	✓	0	✓	0	✓

**✓** indicates the presence and **0** indicates the absence of the correspondent compound.

**Table 2:** Surface properties of biopolymeric and hybrid membranes: wettability (θ_water_), percentage of water absorption (%W_a_), free surface energy (γ_S_), and its dispersive (γ_S_^D^) and polar (γ_S_^P^) components.

Membrane	θwater	γ_S_ (mJ.m^−2^)	γ_S_^P^ (mJ.m^−2^)	γ_S_^D^ (mJ.m^−2^)	Water Absorption (%W_a_)
**(HC + Cht)**	72.38 ± 8.16	36.12 ± 8.45	9.57 ± 5.24	26.56 ± 6.63	143.5 ± 0.6
**(HC + Cht)_HAp**	17.85 ± 1.79	70.47 ± 1.71	61.04 ± 1.6	9.43 ± 0.58	1342.2 ± 0.8
**(HC + Cht)_** **SHAp**	53.62 ± 3.19	53.9 ± 5.94	46.47 ± 5.04	7.02 ± 2.42	160.1 ± 0.3
**(HC + κ-carr)**	58.99 ± 7.66	36.73 ± 8.22	15.96 ± 6.16	20.77 ± 5.44	611.4 ± 0.5
**(HC + κ-carr)_HAp**	17.58 ± 5.35	68.87 ± 3.8	56.33 ± 3.6	12.54 ± 1.23	616.5 ± 0.7
**(HC + κ-carr)_SHAp**	23.58 ± 5.89	65.21 ± 5.24	49.41 ± 4.83	15.8 ± 2.04	733.9 ± 0.6

## Data Availability

The data presented in this study are available on request from the corresponding author.

## Supplementary Materials

The following supporting information can be downloaded at: https://www.mdpi.com/article/10.3390/ijms23137277/s1.
