# Supplementary material for: Synthesis of Antibacterial Hybrid Hydroxyapatite/Collagen/Polysaccharide Bioactive Membranes and Their Effect on Osteoblast Culture
Source: Int J Mol Sci. 2022 Jun 30;23(13):7277. doi: 10.3390/ijms23137277 (PMC9267025; doi:10.3390/ijms23137277)
Supplement: Supplementary file 1 [file ijms-23-07277-s001.zip › ijms-1770080-supplementary.pdf]

Synthesis of antibacterial hybrid hydroxyapatite/collagen/polysaccharide bioactive membranes and their effect on osteoblast culture

Lucas Fabrício Bahia Nogueira[1], Marcos Antônio Eufrásio Cruz[1], Guilherme José Aguilar[1], Delia Rita Tapia Blácido[1], Márcia Eliana da Silva Ferreira[2], Bianca Chieregato Maniglia[1], Massimo Bottini[3], Pietro Ciancaglini[1], Ana Paula Ramos[1]*

[1]*Departamento de Química, Faculdade de Filosofia, Ciências e Letras de Ribeirão Preto -Universidade de São Paulo*
[2]*Departamento de Ciências Farmacêuticas, Faculdade de Ciências Farmacêuticas de Ribeirão Preto -Universidade de São Paulo*
[3]*Department of Experimental Medicine, University of Rome Tor Vergata, Rome, Italy*

*corresponding author: anapr@ffclrp.usp.br

Supplementary material

S.1 Additional mechanical parameters

Additional parameters obtained in the tensile and elongation tests are related to YM. The higher the YM, the higher the resistance of the material to deformations, consequently the lower its elongation and the higher the tension required to deform it.[37] Thus, the hybrid membranes that showed more significant increases in YM in relation to the control, specifically (HC + Cht)_HAp and (HC + κ-carr)_HAp, also exhibited low elongation rate (Figures S1A and S1B) and required higher tension to undergo deformation (Figures S1C and S1D). In the case of (HC + Cht) and (HC + κ-carr), controls, for the same elongation rate, around 27%, the applied stresses were 1.10 $\pm$ 0.11 MPa and 3.7 $\pm$ 0.6 MPa, respectively. This result reflected the organization of the carrageenan chains in a three-dimensional arrangement stabilized by electrostatic interactions.

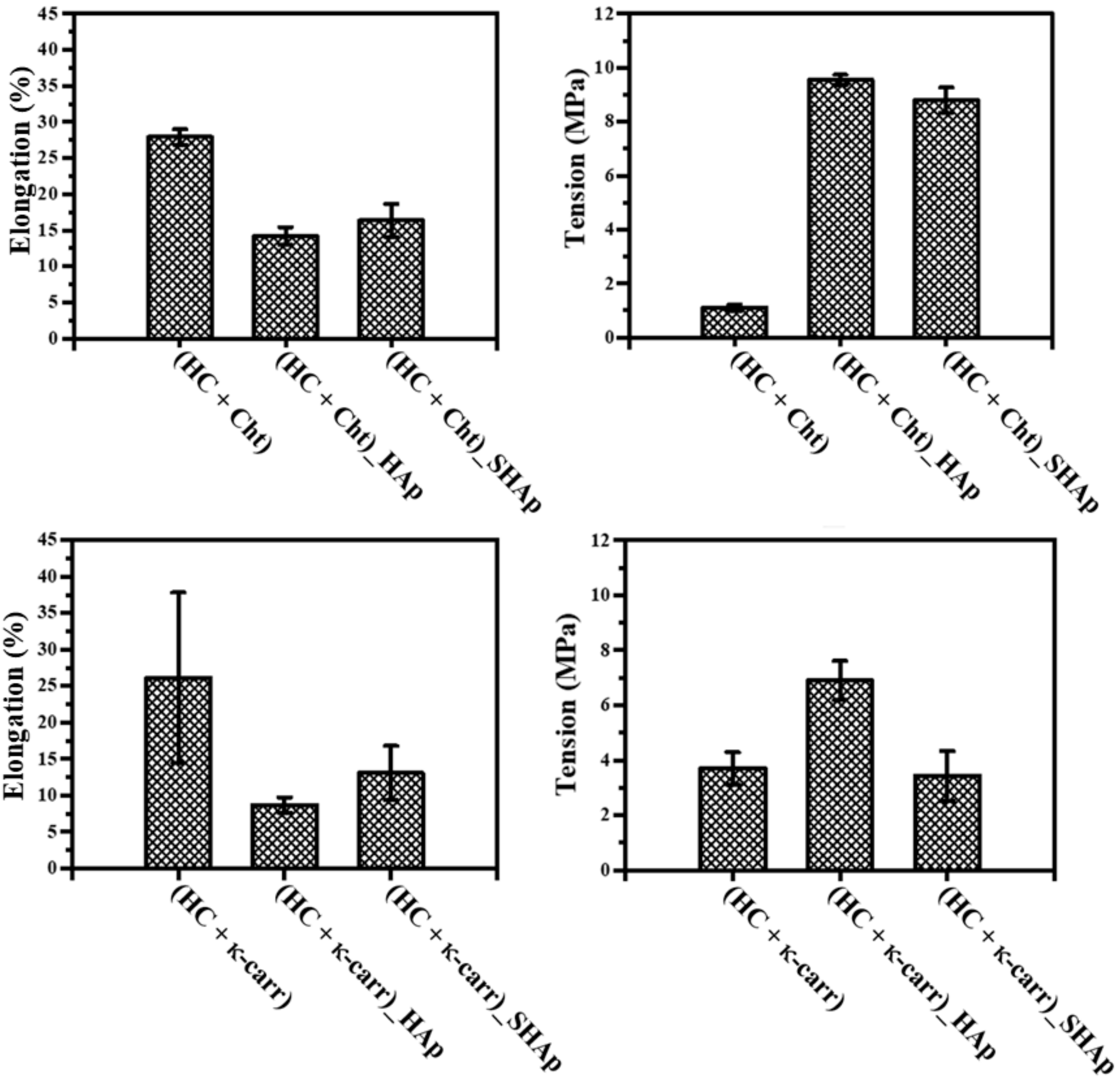

Figure S1. **Comparison of additional mechanical parameters obtained in the tensile tests.** The biopolymeric membranes based on hydrolyzed collagen (HC) and chitosan (Cht) ((HC + Cht), (HC + Cht)_HAp, and (HC + Cht)_SHAp), and hydrolyzed collagen (HC) and κ-carrageenan (κ-carr) ((HC + κ-carr), (HC + κ-carr)_HAp, and (HC + κ-carr)_SHAp) were analyzed with regards to the elongation and tension.

S.2 Bioactivity test using simulated body fluid (SBF)

The diffraction patterns obtained for the hybrid membranes after exposition of the hybrid membranes to SBF depicted in the Figure S2, revealed higher intensity and better definition of the peaks related to the formation of HAp (i.e., $2\theta = 32°$), in special for the samples obtained by methodology 1 (Figure S2A and Figure S2B). Moreover, the relative intensity of the peaks related to the formation of brushite

compared to HAp was reduced after the immersion of the membrane (HC + Cht)_SHAp into SBF (Figure S2C).

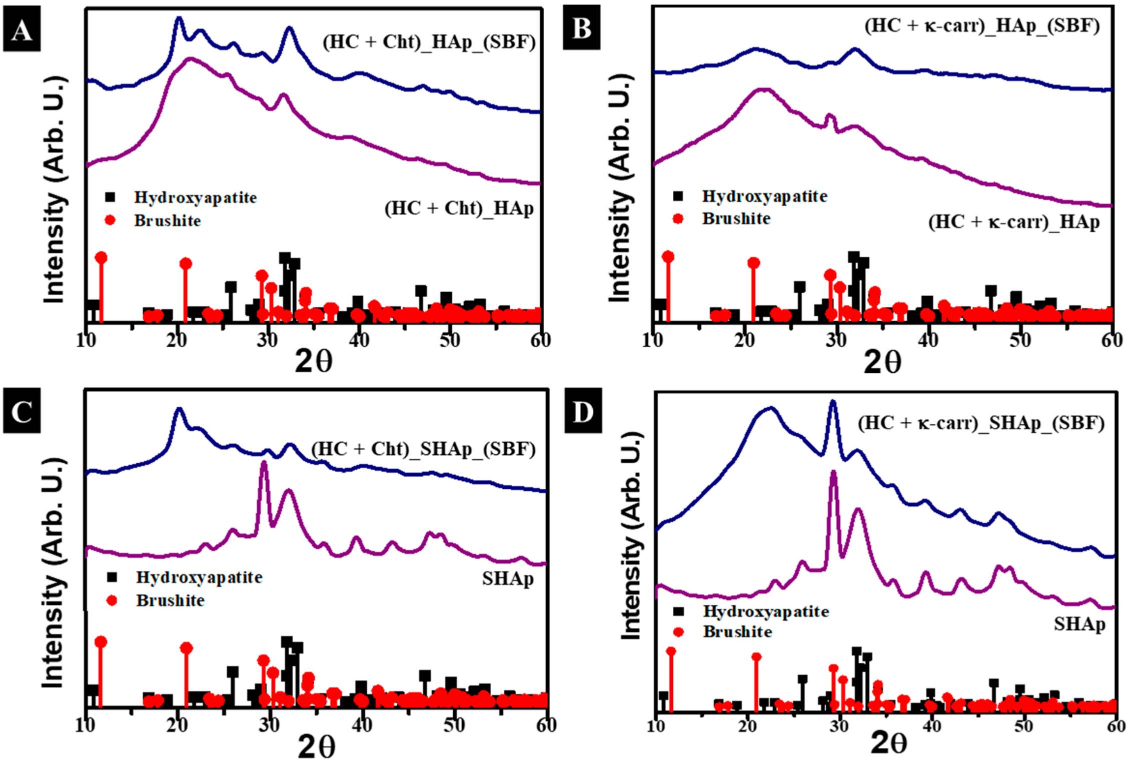

Figure S2. **Characterization of the membranes after the bioactivity test carried out by immersion in SBF.** Comparison between the X-ray diffraction patterns obtained before (purple line) and after (blue line) exposed to SBF, for 30 minutes, the HC-based membranes mixed with A, C) chitosan (Cht) and B, D) κ-carrageenan (κ -carr), with the incorporation of mineral phase by methodology 1 (A, B) and 2 (C, D). The mineral phases were identified by the diffraction patterns (■) 9011092- hexagonal hydroxyapatite and (●) 1533075- monoclinic brushite from the Crystallography Open Database (COD).

S.3 Characterization of the silver nanoparticles (AgNP)

Figure S3A shows the UV-Vis spectrum obtained for the AgNP colloidal dispersion. An absorption band at 390 nm emerged, which is characteristic of spherical AgNP [120,121] and is related to collective oscillation of free electrons on the surface of the metallic particle due to interaction with the electric field of light. In other words, this band corresponded to a plasmonic resonance band.[122]

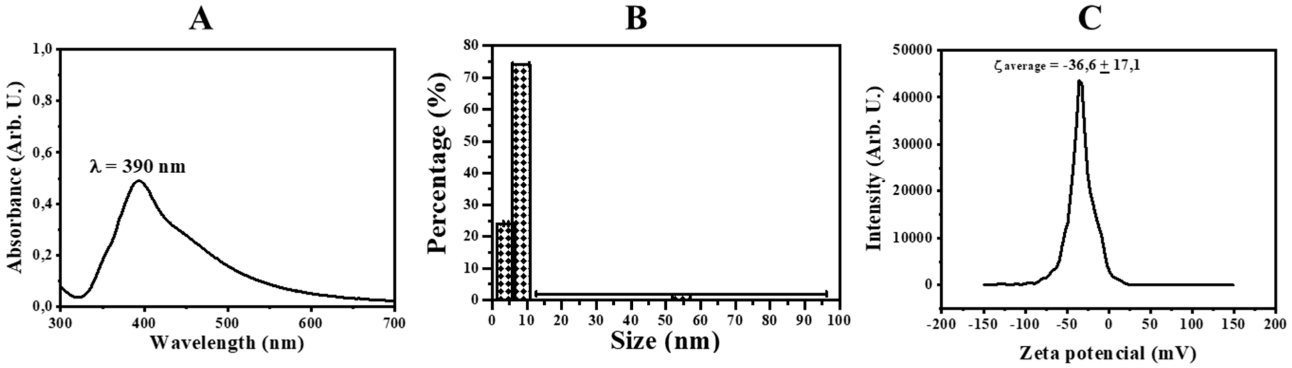

Figure S3. **Characterization of the silver nanoparticles (AgNP).** (A) UV-Vis spectrum, (B) particle size distribution, and (C) zeta potential measurement (ζ) of the colloidal AgNP dispersion.

Regarding size distribution, Figure S3B revealed non-homogeneity and that approximately 74% of the particles ha average size of 8.2 nm, while 24% of the particles had average size of 3.8 nm. These values were close to the values reported in the literature when 2 mmol.L^{-1} $AgNO_3$ was used in the synthesis.[123] The zeta potential determined for these particles was -36 ± 17 mV (Fig. S3C), indicating high colloidal stability. The AgNP negative potential was due to adsorption of BH_4^- ions resulting from $AgNO_3$ chemical reduction with sodium borohydride on the surface of the nanoparticles, which stabilized them electrostatically.

S.4 AgNP release from hybrid membranes

Table S3 shows the AgNP release rate, which was monitored by absorption spectrometry in the Ultraviolet-Visible region after immersion of the hybrid membranes containing 2.5 wt.% HC and 2.5 wt.% polysaccharides, Cht or κ-carr, and HAp obtained by Methodology 1 after immersion in in water at 37 °C for 1, 3, 5, 18, 20, and 24 h.

Table S3 - AgNP concentration released by the hybrid membranes during immersion in water at 37 °C

Equation: $y = 35425x + 0.0065$ (R^2=0.99979998)				
**(HC + Cht)_HAp**				
**Time (H)**	**Absorbance at 390 nm**	**AgNp concentration released ($mol.L^{-1}$)**	**AgNp concentration released ($mg.cm^{-2}$)**	**Release rate (%)**
**1**	0.00704	$1.5244 \cdot 10^{-8}$	$2.4695 \cdot 10^{-6}$	0.0049
**3**	0.03077	$6.8519 \cdot 10^{-7}$	$1.1100 \cdot 10^{-4}$	0.2220
**5**	0.03992	$9.4329 \cdot 10^{-7}$	$1.5281 \cdot 10^{-4}$	0.3056
**18**	0.11139	$2.9609 \cdot 10^{-6}$	$4.7966 \cdot 10^{-4}$	0.9593
**20**	0.14879	$4.0167 \cdot 10^{-6}$	$6.5070 \cdot 10^{-4}$	1.3014
**24**	0.19935	$5.4439 \cdot 10^{-6}$	$8.8191 \cdot 10^{-4}$	1.7638
**(HC + κ-carr)_HAp**				
**Time (H)**	**Absorbance at 390 nm**	**Released AgNP concentration ($mol.L^{-1}$)**	**Released AgNP concentration ($mg.cm^{-2}$)**	**Release rate (%)**
**1**	0.183	$4.9824 \cdot 10^{-6}$	0.0008	1.6142
**3**	0.26155	$7.1997 \cdot 10^{-6}$	0.0012	2.3327
**5**	0.36304	$1.0065 \cdot 10^{-5}$	0.0016	3.2610
**18**	0.69551	$1.9450 \cdot 10^{-5}$	0.0032	6.3018
**20**	0.70574	$1.9739 \cdot 10^{-5}$	0.0032	6.3954
**24**	0.95999	$2.6916 \cdot 10^{-5}$	0.0044	8.7208
